# Obesity and survival among a cohort of breast cancer patients is partially mediated by tumor characteristics

**DOI:** 10.1038/s41523-019-0128-4

**Published:** 2019-10-02

**Authors:** Cindy K. Blair, Charles L. Wiggins, Andrea M. Nibbe, Curt B. Storlie, Eric R. Prossnitz, Melanie Royce, Lesley C. Lomo, Deirdre A. Hill

**Affiliations:** 1 Department of Internal Medicine, University of New Mexico, Albuquerque, NM USA; 2 University of New Mexico Comprehensive Cancer Center, Albuquerque, NM USA; 3 Department of Biomedical Statistics and Informatics, Mayo Clinic College of Medicine, Rochester, MN USA; 4 Department of Pathology, University of Utah, Salt Lake City, UT USA

**Keywords:** Breast cancer, Cancer epidemiology

## Abstract

Obesity exerts adverse effects on breast cancer survival, but the means have not been fully elucidated. We evaluated obesity as a contributor to breast cancer survival according to tumor molecular subtypes in a population-based case–cohort study using data from the Surveillance Epidemiology and End Results (SEER) program. We determined whether obese women were more likely to be diagnosed with poor prognosis tumor characteristics and quantified the contribution of obesity to survival. Hazard ratios (HRs) and 95% confidence intervals (CI) were calculated via Cox multivariate models. The effect of obesity on survival was evaluated among 859 incident breast cancers (subcohort; 15% random sample; median survival 7.8 years) and 697 deaths from breast cancer (cases; 100% sample). Obese women had a 1.7- and 1.8-fold increased risk of stage III/IV disease and grade 3/4 tumors, respectively. Obese women with Luminal A- and Luminal B-like breast cancer were 1.8 (95% CI 1.3–2.5) and 2.2 (95% CI 0.9–5.0) times more likely to die from their cancer compared to normal weight women. In mediation analyses, the proportion of excess mortality attributable to tumor characteristics was 36.1% overall and 41% and 38% for Luminal A- and Luminal B-like disease, respectively. Obesity was not associated with breast cancer-specific mortality among women who had Her2-overexpressing or triple-negative tumors. Obesity may influence hormone-positive breast cancer-specific mortality in part through fostering poor prognosis tumors. When tumor biology is considered as part of the causal pathway, the public health impact of obesity on breast cancer survival may be greater than previously estimated.

## Introduction

Currently in the U.S., almost 40% of the adult population is obese, and another 30% is overweight.^1,2^ Obesity is one of the most prevalent modifiable risk factors for chronic disease and has received considerable attention in relation to cancer outcomes. However, despite numerous studies that have evaluated obesity and breast cancer-specific mortality, the means through which obesity exerts its effects on breast cancer survival have not been fully elucidated.

In recent meta-analyses, breast cancer-specific death among obese women is elevated 1.3-fold compared to normal weight women.^3,4^ However, in most studies, risk estimates for the relationship between body mass index (BMI) and breast cancer mortality have been adjusted for tumor characteristics (stage at diagnosis and/or tumor size, grade, and nodal status). Evidence is accumulating that tumor characteristics may be on the causal pathway (potential mediators) between BMI and cancer mortality.^5–7^ If so, then the BMI–mortality risk estimates adjusted for these factors is likely to be underestimated. Statistical adjustment for a causal intermediate can substantially attenuate risk estimates or even result in a reversal of direction (suggested reduced risk).^8^

In a large population-based case–cohort study, we investigated the role of obesity and associated mechanisms on breast cancer-specific death. We first examined whether higher BMI was associated with more aggressive tumor characteristics. We then investigated the relationship between higher BMI and breast cancer survival, with consideration of the possibility that tumor characteristics were on the causal pathway, often termed “mediators.” We evaluated the BMI–mortality relationship according to breast cancer subtypes, as results from previous studies have suggested that the effect of BMI may be stronger in luminal disease.^9–14^

## Results

### Population characteristics

**Fig. 1:**
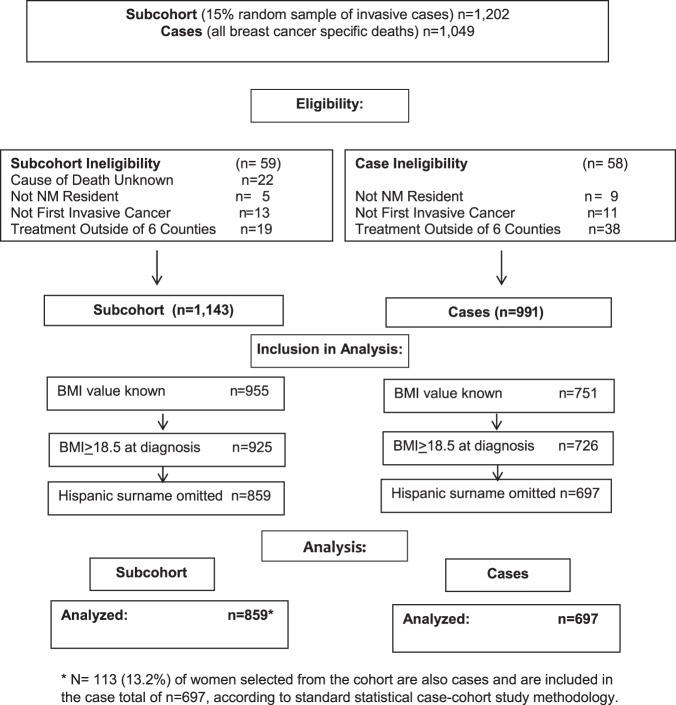
Study flow diagram of a population-based case–cohort study, including cases (all deaths due to breast cancer-related causes) and a subcohort (a 15% random sample of all other eligible breast cancers) that was used to examine the relationship between body mass index and breast cancer-specific mortality

The analytic case–cohort consisted of 697 deaths from breast cancer (cases) and 859 breast cancers (subcohort; 15% random sample weighted by 6.67 × (1/sampling fraction) in the analysis) (Fig. 1). Median follow-up in the subcohort was 94 months (range 2–205 months). Women who died from breast cancer were more likely to be Hispanic/Latina (33% vs. 23%), obese (33% vs. 24%), and to be diagnosed with later stage tumors (stage III/IV; 50% vs. 17%) as well as more aggressive tumor subtypes (non-Luminal A like; 40% vs. 26%), compared to the subcohort (Table 1—before imputation; Supplementary Table 1—after imputation).

**Table 1:** Characteristics of included women diagnosed with incident invasive breast cancer in six New Mexico counties

Characteristic	Subcohort (*N* = 859) 15% random sample		Breast cancer deaths (*N* = 697) 100% sample	
	N	(%)	N	(%)
Age at diagnosis (years)				
<40	43	5.0	56	8.1
40–49	146	17.0	143	20.5
50–59	217	25.2	180	25.8
60–69	206	24.0	117	16.8
70–79	158	18.4	123	17.6
80+	89	10.4	78	11.2
Year of diagnosis				
1997–2000	253	29.4	257	36.9
2001–2004	256	29.8	238	34.1
2005–2009	350	40.8	202	30.0
Race/ethnicity				
Non-Hispanic white	661	76.9	464	66.6
Hispanic/Latina	198	23.1	233	33.4
Menopausal status^a^				
Pre/peri	160	18.6	168	24.1
Postmenopausal	666	77.5	497	71.3
Missing	33	6.2	32	4.6
Body mass index (kg/m^**2**^)^a^				
18.5–24.9	338	39.3	252	36.2
25.0–29.9	246	28.6	172	24.7
>30.0	195	22.7	211	30.3
Missing	80	9.3	62	8.9
Stage^a^				
1	382	44.5	94	13.5
2	281	32.7	222	31.9
3/4	133	15.5	307	44.0
Missing	63	7.3	74	10.6
Tumor subtype^a^				
Luminal A	526	61.2	320	45.9
Luminal B	91	10.6	105	15.1
Her2+ ER−/PR−	24	2.8	32	4.6
Triple negative	61	7.1	85	12.2
Missing	157	18.3	155	22.2
Treatment (yes)^b^				
Chemotherapy	381	44.4	469	67.3
Radiation	560	65.2	400	57.4
Endocrine therapy	586	68.2	418	60.0

*ER* estrogen receptor, *Her2* human epidermal growth factor receptor 2, *PR* progesterone receptor, *Pre/peri* premenopausal/perimenopausal

^a^The table reflects the frequencies prior to imputation, thus missing data are shown, which reflect the number of imputed values for each variable

^b^Non-exclusive categories

### Mediation analysis

Tumor characteristics differed by BMI (Table 2). Women who were obese were more likely to be diagnosed with stage 3/4 disease, and particularly, metastases, as well as grade 3/4 tumors, relative to normal weight women. Obese women were also 2-fold more likely to have ≥2 comorbidities and 1.5-fold more likely to receive chemotherapy than normal weight women.

**Table 2:** Overweight and obesity in relation to tumor characteristics, Charlson comorbidity index, and treatment: cross-sectional analysis in a population-based subcohort (*n* = 859)

	Normal^a^	Overweight^a^	Obese^a^	Overweight^b^	Obese^b^
	*N* (%)	*N* (%)	*N* (%)	Odds ratio (95% CI)	Odds ratio (95% CI)
Stage					
1	197 (52.3)	125 (45.8)	88 (42.1)	1.00 (reference)	1.00 (reference)
2	129 (34.2)	99 (36.3)	78 (25.5)	1.17 (0.79–1.73)	1.30 (0.86–1.95)
3/4	51 (13.5)	49 (18.9)	43 (20.6)	1.46 (0.89–2.40)	1.73 (1.02–2.93)
*p* trend				0.10	0.01
Components of stage					
Tumor size, cm					
<2	235 (62.3)	154 (56.4)	117 (56.0)	1.00 (reference)	1.00 (reference)
2–5	108 (28.7)	98 (35.9)	62 (29.7)	1.45 (0.99–2.11)	1.25 (0.82–1.89)
5+/chest wall/skin	34 (9.0)	21 (7.7)	30 (14.4)	0.98 (0.53–1.80)	1.75 (0.99–3.10)
*p* trend				0.20	0.05
Lymph nodes					
0	249 (66.1)	166 (60.8)	127 (60.8)	1.00 (reference)	1.00 (reference)
1–3	84 (22.3)	69 (25.3)	51 (24.4)	1.21 (0.78–1.85)	1.15 (0.73–1.82)
4+	44 (11.7)	38 (13.9)	31 (14.8)	1.16 (0.66–2.00)	1.51 (0.85–2.67)
*p* trend				0.39	0.16
Metastasis					
No	367 (97.1)	265 (97.1)	199 (95.2)	1.00 (reference)	1.00 (reference)
Yes	7 (1.9)	8 (2.9)	10 (4.8)	2.05 (0.56–7.52)	3.33 (1.00–11.22)
Grade					
1	123 (33.5)	93 (34.3)	46 (22.4)	1.00 (reference)	1.00 (reference)
2	139 (37.9)	104 (38.4)	90 (43.9)	1.06 (0.70–1.60)	1.77 (1.11–2.80)
3/4	105 (28.6)	74 (27.3)	69 (33.7)	0.99 (0.64–1.53)	1.79 (1.10–2.91)
*p* trend				0.99	0.02
Charlson comorbidity score					
0	298 (79.1)	208 (76.2)	130 (62.2)	1.00 (reference)	1.00 (reference)
1	35 (9.3)	25 (9.2)	43 (20.6)	0.96 (0.53–1.73)	2.74 (1.61–4.68)
≥2	44 (11.6)	40 (14.6)	36 (17.2)	1.23 (0.74–2.03)	2.02 (1.21–3.40)
*p* trend				0.30	<0.01
Treatment received					
Chemotherapy					
No	269 (71.4)	185 (67.8)	132 (63.2)	1.00 (reference)	1.00 (reference)
Yes	108 (28.7)	88 (32.2)	77 (36.8)	1.27 (0.86–1.86)	1.48 (0.99–2.21)
Radiation					
No	210 (55.7)	154 (56.4)	109 (52.2)	1.00 (reference)	1.00 (reference)
Yes	167 (44.3)	119 (43.6)	100 (47.8)	1.08 (0.75–1.55)	0.84 (0.57–1.23)
Endocrine^c^					
No	263 (69.8)	175 (64.1)	135 (64.6)	1.00 (reference)	1.00 (reference)
Yes	114 (30.2)	98 (35.9)	74 (35.4)	1.29 (0.79–1.95)	0.78 (0.49–1.26)

*CI* confidence interval

^a^Normal weight = 18.5–24.9 kg/m^2^; overweight = 25.0–29.9 kg/m^2^; obese = ≥ 30 kg/m^2^

^**b**^Adjusted for age at diagnosis (10-year age categories)

^**c**^Among estrogen receptor (ER)-positive tumors only

**Fig. 2:**
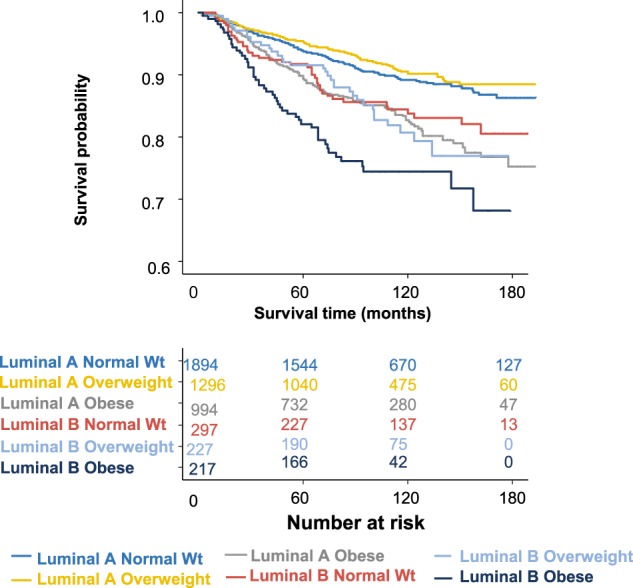
Breast cancer-specific survival by body mass index categories for Luminal A-like and Luminal B-like tumor subtypes (adjusted for age at diagnosis)

Overweight women did not have an elevated risk of breast cancer-specific mortality. In contrast, obese women had 1.63-fold overall increased risk (Table 3—HR1), compared to normal weight women. Obese women also had a 1.78–2.16-fold increased breast cancer mortality risk within subgroups of Luminal A- and Luminal B-like disease, respectively (Table 3—HR1; Fig. 2). However, there was little evidence for an association between obesity and mortality among women who had Her2-overexpressing or triple-negative tumors. Premenopausal obese women had statistically non-significantly elevated hazard ratios (HRs) for breast cancer mortality in luminal tumors than postmenopausal obese women, but mortality was not elevated for non-luminal tumors in either menopausal status group (Supplementary Table 2). The effects of obesity and luminal vs. non-luminal subtype on breast cancer survival did not exceed that expected from their joint multiplicative effects (*p* value for interaction = 0.08).

**Table 3:** Body mass in relation to breast cancer-specific mortality by tumor subtype

BMI category^a^	Cohort, *N* = 859; *N* (%)	Deaths, *N* = 697; *N* (%)	HR1 (95% CI)^b^	HR2 (95% CI)^c^	Mediation proportion
Overall					
Normal weight	377 (43.9)	279 (40.0)	1.00	1.00	
Overweight	273 (31.8)	185 (26.5)	0.98 (0.76–1.27)	0.86 (0.61–1.20)	
Obese	209 (24.3)	233 (33.4)	1.63 (1.26–2.11)	1.33 (0.95–1.84)	36.1%
Luminal A subtype					
Normal weight	286 (44.8)	166 (40.7)	1.00	1.00	
Overweight	203 (31.8)	102 (25.0)	0.94 (0.67–1.32)	0.76 (0.50–1.16)	
Obese	150 (23.4)	140 (34.3)	1.78 (1.28–2.48)	1.41 (0.91–2.18)	41.4%
Luminal B subtype					
Normal weight	44 (41.1)	44 (34.4)	1.00	1.00	
Overweight	32 (29.9)	34 (26.6)	1.27 (0.51–3.13)	1.36 (0.41–4.57)	
Obese	31 (29.0)	50 (39.0)	2.16 (0.93–4.99)	1.62 (0.49–5.33)	37.7%
Her2-overexpressing subtype					
Normal weight	14 (46.7)	25 (52.1)	1.00		
Overweight	9 (30.0)	10 (20.8)	0.60 (0.10–3.45)	–	–
Obese	7 (23.3)	13 (27.1)	1.09 (0.14–8.84)		
Triple-negative subtype					
Normal weight	33 (39.8)	44 (38.9)	1.00	1.00	
Overweight	29 (34.9)	39 (34.5)	1.23 (0.55–2.74)	1.32 (0.46–3.80)	–
Obese	21 (25.3)	30 (26.6)	1.18 (0.54–2.57)	1.04 (0.42–2.54)	

*CI* confidence interval, *Her2* human epidermal growth factor receptor 2, *HR* hazard ratio, — not calculated due to small cell sizes

^a^Normal weight = 18.5–24.9 kg/m^2^; overweight = 25.0–29.9 kg/m^2^; obese = ≥ 30 kg/m^2^

^b^HR1—Adjusted for age (10-year age groups) and Hispanic ethnicity

^c^HR2—Adjusted for age (10-year age groups), Hispanic ethnicity, stage at diagnosis (I, II, III, or IV), and tumor grade (1, 2, 3/4)

Statistical adjustment for tumor characteristics substantially reduced the relationship between BMI and mortality for luminal tumors (Table 3—HR2). This suggests that these factors have a limited influence on the BMI–mortality relationship after accounting for tumor stage and grade. The proportion of the obesity–mortality relationship mediated by poor prognosis tumor characteristics was 41.4% for Luminal A-like and 37.7% for Luminal B-like subtypes (see p. 493 in ref. ^15^). Thus the age- and ethnicity-adjusted model (Table 3—HR1) provides an unbiased estimate of the BMI effect on breast cancer mortality, unadjusted for factors that appear to mediate such effects.

## Discussion

Our large, population-based case–cohort study afforded the opportunity to investigate the means whereby BMI exerts influence on breast cancer-specific mortality. Obesity appears to influence breast cancer survival in part by fostering greater tumor size, higher grade, and a substantially increased risk of metastases. We found that 36% of the increased mortality risk overall due to obesity was potentially attributable to (or mediated by) tumor characteristics at diagnosis and 38–41% for luminal subtypes. Thus the proportion of the effect of obesity on breast cancer survival that acts through (is mediated by) tumor biology is high.

To date, the increased mortality risk in obese women has been identified predominantly in ER+/luminal subtypes,^9–14^ consistent with our findings. Relative risk estimates in those studies have ranged from 1.2–1.5, lower than the 1.7–1.8-fold we report. This difference, albeit modest, is possibly in part due to attenuation via adjustment for tumor characteristics (HR2, Table 3) or other potential mediators. Our results contrast somewhat with studies in which obesity-associated mortality risk was elevated in women with triple-negative tumors.^16–19^ Of note, those studies included all-cause mortality as a component or sole endpoint. BMI is expected to be related to all-cause mortality in part due to cardiovascular and other chronic disease mortality even if minimal or no relationship with breast cancer-specific survival.

Our results should be interpreted in light of study limitations and strengths. The smaller sample size constrained our ability to provide precise effect estimates for non-luminal subtypes. Weight loss at diagnosis, especially if non-intentional, could have influenced our results. Strengths of our population-based study include a case–cohort design with nearly 700 deaths from breast cancer, facilitating risk estimates for Luminal A and Luminal B breast cancer subtypes.

The findings position obesity as a potential driver of poor prognosis tumor biology. Our results imply that excess body weight may contribute to higher tumor stage and grade, including a greater incidence of metastases, specifically in the ~80% of breast tumors that are considered luminal. Obese women have had aggressive tumor characteristics in previous investigations^13,19–23^; however, such factors have been considered confounders and not identified as intermediates on the causal pathway and thus strong mediators of disease outcomes. Such an analysis will attenuate measures of clinical and public health impact, which is of concern because obesity may be one of few modifiable risk factors for breast cancer prognosis. Whether weight loss after a diagnosis has beneficial effects on cancer outcomes is unknown and may depend in part on whether the direct and indirect effects of obesity exposure are reversible. Currently, several randomized controlled trials are evaluating the effects of weight loss among breast cancer survivors on cancer recurrence and long-term outcomes.^24,25^

Previous and current evidence linking obesity and poor prognosis tumor characteristics provide a foundation for understanding tumor characteristics as mediators of the BMI–breast cancer survival relationship. Biological mechanisms linking excess adiposity to poor prognosis tumor characteristics include local and systemic alterations to inflammatory markers, steroid hormones, cytokines/adipokines, insulin, and insulin-like growth factors (IGFs). The local and systemic alterations may directly affect cancer cells via activation of signaling pathways (e.g., estrogen, insulin/IGF, Janus-activated kinase/signal transducer and activator of transcription factor, etc.) or alterations in cellular metabolism or can indirectly affect the tumor microenvironment to promote cell proliferation, angiogenesis, and invasion.^26–28^ In addition, obese women have had greater tumor proliferation in several studies.^21,29,30^ Such data do not establish that BMI contributes to adverse tumor characteristics directly. Alternative explanations for poor prognosis tumor characteristics include the possibility that initial cancer detection is delayed in obese women^31–33^ and that delayed detection of recurrence and metastases further contribute to survival, but that does not account for the restriction of many findings, including ours, to luminal tumors.

The lack of association between obesity and survival of non-luminal cancers is puzzling, as such tumors are more likely to have a poor prognosis. Despite being one of the larger population-based studies to examine obesity and breast cancer-specific mortality, assessment of some relationships with non-luminal tumors were limited by cell sizes in this study. However, local and systemic tissue-specific mechanisms that link obesity with progression and survival may plausibly support greater growth and proliferation in hormone receptor-positive breast cancers. The occurrence and severity of breast adipose tissue inflammation is higher in obese compared to normal weight women^34^ and in postmenopausal relative to premenopausal women.^35,36^ Breast adipose tissue inflammation is associated with elevated aromatase levels, the rate-limiting enzyme in estrogen biosynthesis,^37^ and similar inflammation has been noted at distant sites.^38,39^ Thus increased estrogen production arising from aromatase activity, particularly in the postmenopausal environment, may selectively drive proliferation in estrogen-dependent breast cancer. Biosynthesis of estrogen may also reduce the effectiveness of endocrine therapy among obese women with hormone-positive subtypes.^37^ These cellular processes lend support to the dichotomy between luminal and non-luminal disease evident in this study. Less is known regarding other possible mechanisms linking obesity to progression and survival of non-hormone-related subtypes; however, this is an active area of research.

Whether poorer prognosis tumor characteristics are an effect of adiposity, delayed diagnosis, metabolic changes, or other or combined mechanisms, the clinical effect of obesity will be attenuated by statistical adjustment for factors that are a consequence of exposure. Thus the effect of BMI on breast cancer outcomes is likely to be somewhat stronger than previously reported. Our findings that BMI may influence tumor characteristics have implications for tumors beyond breast cancer.

## Methods

### Study design and population

Our population-based case–cohort study of contributors to breast cancer survival has been previously described.^40,41^ Eligible were Hispanic white and non-Hispanic white women diagnosed with invasive breast cancer between 1997 and 2009 in six New Mexico counties (representing 50% of the New Mexico population). Breast cancer cases were identified through the New Mexico Tumor Registry, a founding member of the Surveillance, Epidemiology, and End Results program. To capture complete treatment information, only women who received treatment within the six county geographic area were eligible. Hispanic ethnicity was classified according to the North American Association of Central Cancer Registries algorithm. Cases identified solely by autopsy or death certificate and women with Hispanic ethnicity defined only by surname were excluded. The study population included all deaths attributed to breast cancer as an underlying cause on the death certificate, and a 15% random sample of all eligible breast cancers. Analytic models were restricted to women with BMI ≥ 18.5 kg/m^2^ (Fig. 1).

### Exposure, covariates, and outcome

Height and weight at clinical visits were abstracted from medical charts. Weight at diagnosis was available for 91.6% of women, prior to diagnosis only for 0.6% of women, and after diagnosis only for 7.8% of women. Missing weight at diagnosis was imputed for women with at least one other weight measurement (see “Statistical analyses” below).^42^ Weight and height were used to calculate BMI at diagnosis, which was categorized as normal weight (18.5–24.9 kg/m^2^), overweight (25–29.9 kg/m^2^), and obese (≥30.0 kg/m^2^).^43^ Small cell sizes precluded examination of higher obesity levels (e.g., ≥35.0) and underweight (BMI < 18.5 kg/m^2^). Women completely missing height or at least a single weight in the medical records (*N* = 240 cases; 188 subcohort) were excluded from analyses. Tumor size, node status, and metastases, tumor grade, treatment information,^41^ and Charlson comorbidities^44^ were abstracted from paper and electronic medical records. Tumor stage was categorized according to the American Joint Committee on Cancer V.6.

Biological markers determined by immunohistochemistry (IHC) including estrogen receptor (ER), progesterone receptor (PR), and Her2/neu (confirmed by fluorescent in situ hybridization) were abstracted from diagnostic pathology reports. IHC results were also obtained from tissue microarrays (TMAs) stained for ER, PR, and Her2/neu when missing. Women were considered ER or PR positive if >1% IHC staining was present^45^ and Her2 positive if staining intensity was >=3+. Remaining values for women not included in TMAs (ER 6.7%, PR 12.6%, and Her2 24.7%) were imputed.^42^ Breast cancer subtypes were categorized as: Luminal A like (ER or PR positive, Her2 negative), Luminal B like (ER or PR positive, Her2 positive), Her2 overexpressing (ER and PR negative, Her2 positive), and triple negative (ER, PR, and Her2 negative).^18,46,47^

The primary outcome of death from breast cancer was identified by the New Mexico Tumor Registry through linkages with the New Mexico Bureau of Vital Records and Health Statistics and the National Death Index. Underlying cause of death was coded using the International Classification of Diseases (ICD) Ninth and Tenth edition codes (ICD-9: 174 or ICD-10: C50.1–C50.9).

### Multiple imputation methods

The sequence of regression models approach was used to impute missing values for all variables except body weight.^42^ A separate model was formulated for each variable, starting with an unconditional (mean only) model for the variable with the least missing and ending with a model for the variable with the most missing values, conditional on all other variables. (Fully imputed values are provided in Supplementary Table 1.) Variables used in the imputation included age at diagnosis, ethnicity, height, tumor size, lymph node status, tumor grade, ER, PR, Her2/neu, menopausal status, marital status, census tract income and education, receipt of chemotherapy, radiation and endocrine therapy, Charlson comorbidity conditions,^44^ vital status, and survival time.

For imputation of missing weight at diagnosis from other weight measurement(s), additional modeling took into account the timing of the additional measures. A subject’s weight was allowed to vary over time according to an Ornstein–Uhlenbeck (O-U) process with subject-specific mean level as a function of available covariates. The O-U process was evaluated on a month grid, using observed weight when available, thus making it equivalent to an autoregressive (AR(1)) model.^48^ Estimation and imputation for all variables were carried out simultaneously via a Bayesian computational framework (rjags package in R). To assess the performance of the weight imputation model, a cross-validation was run by predicting weight at diagnosis for 20% of women with a known weight. *R*-squared for the resulting prediction was 82.9.

### Statistical analyses

Statistical analyses were conducted using the standard case–cohort methodology.^49^ Women in the subcohort were weighted by the inverse of the sampling fraction (100%/15% = 6.67). Multivariate Cox proportional hazards models were used to estimate HRs and 95% confidence intervals. The time scale was time in months from breast cancer diagnosis until date of last follow-up, death, or January 1, 2013. Breast cancer-specific death was the outcome of interest. All other causes of death or women alive at last follow-up were censored. Associations between BMI and breast cancer mortality were stratified by tumor molecular subtypes. Diagnosis age (<40, 40–49, 50–59, 60–69, 70–79, ≥80 years) and Hispanic ethnicity were selected a priori to be included in regression models. There were no substantial changes to the estimates when age was included as a continuous variable in the model, thus results are based on the model including diagnosis age as a categorical variable. Linear trend was calculated by examining categorical variables as ordinal in the model.

To assess tumor characteristics as mediators of the BMI–survival relationship, we first examined whether higher BMI was associated with more aggressive tumor characteristics, which suggests that tumor features might be consequences of BMI. Specifically, we determined whether tumor stage and tumor grade, rather than being evaluated as potential confounders, might more appropriately be considered intermediate variables on the causal pathway (sometimes termed causal mediators) between BMI and mortality. As evidence, we assessed the temporal relationship (BMI exposure precedes each mediator), dose–response, and specificity of the relationship. We examined the cross-sectional association between BMI and potential mediators in polytomous logistic regression models, including only the population-based subcohort, adjusted for diagnosis age. Findings from these investigations were used to inform the analysis of the role of BMI in breast cancer survival.

Cox proportional hazards models for breast cancer-specific mortality were fit for the case–cohort to quantify the effect of BMI on mortality potentially mediated through tumor characteristics. We first calculated the age-adjusted association between BMI and mortality. Then we added tumor characteristics to the model. The mediation proportion, calculated using the difference in log-hazards (see p. 493 in ref. ^15^), suggests the proportion of the BMI effect on mortality that acts through potential intermediates, such as tumor stage and grade.

We evaluated breast cancer-specific mortality within strata of tumor subtypes. We also evaluated whether the effect of BMI on breast cancer-specific mortality differed by subtype, by including the main effects (BMI and luminal vs. non-luminal subtype) and an interaction term (BMI × luminal subtype) in the Cox regression model.

The proportional hazards assumption was validated using Schoenfeld residuals.^50^ Confounding assumptions necessary for causal interpretation of the direct and indirect effect estimates were assessed.^51^ Analyses were conducted using SAS (version 9.4; Cary, N.C.) and R (v.3.4.3, Vienna, Austria). Final multiple imputation estimates were produced using SAS Proc MIanalyze (20 imputations). Kaplan–Meier plots were produced in the R software. A two-sided test of statistical significance was defined as *p* < 0.05. A Health Insurance Portability and Accountability Act waiver of consent was obtained for previously collected data. All study procedures were approved by the University of New Mexico Health Sciences Center institutional review board. Details regarding access to the data supporting this manuscript have been published.^52^

### Reporting summary

Further information on research design is available in the Nature Research Reporting Summary linked to this article.

## Supplementary information

Supplementary Material

Reporting Summary Checklist

## Data Availability

Aggregated patient data supporting all the figures, tables, and supplementary files in the published article are not publicly available to protect patient privacy but will be made available on request from the corresponding author, upon institutional review board approval as described at 10.6084/m9.figshare.9428555.^52^ Release of vital status data is governed by the New Mexico state law, and therefore these data are not currently available. The data generated and analyzed during this study are described in the following data record: 10.6084/m9.figshare.9428555.^52^

## Code availability

Statistical code for imputation methods will be available in the pending papers.
