# Supplementary material for: Obesity and survival among a cohort of breast cancer patients is partially mediated by tumor characteristics
Source: NPJ Breast Cancer. 2019 Oct 2;5:33. doi: 10.1038/s41523-019-0128-4 (PMC6775111; doi:10.1038/s41523-019-0128-4)
Supplement: Supplementary file 1 — Supplementary Material [file 41523_2019_128_MOESM1_ESM.pdf]

Supplemental Table 1. Characteristics of included women diagnosed with incident invasive breast cancer in six New Mexico counties (after imputation).

Characteristic	Subcohort (N=859) 15% random sample N	(%)	Breast Cancer Deaths (N=697) 100% sample N	(%)
**Age at diagnosis (years)**				
<40	43	5.0	56	8.1
40-49	146	17.0	143	20.5
50-59	217	25.2	180	25.8
60-69	206	24.0	117	16.8
70-79	158	18.4	123	17.6
80+	89	10.4	78	11.2
**Year of diagnosis**				
1997 – 2000	253	29.4	257	36.9
2001 – 2004	256	29.8	238	34.1
2005 – 2009	350	40.8	202	30.0
**Race/Ethnicity**				
Non-Hispanic White	661	76.9	464	66.6
Hispanic / Latina	198	23.1	233	33.4
**Menopausal status***				
Pre/Peri	192	22.4	198	28.4
Postmenopausal	667	77.6	499	71.6
**Body Mass Index (kg/m^2)[†]**				
18.5-24.9	377	43.9	279	40.0
25.0-29.9	273	31.8	185	26.5
≥30.0	209	24.3	233	33.4
**Stage[‡]**				
1	410	47.7	101	14.5
2	306	35.6	247	35.4
3/4	143	16.7	349	50.1
**Tumor Subtype[δ]**				
Luminal A	639	74.4	408	59.5
Luminal B	107	12.5	128	18.4
Her2+ ER-/PR-	30	3.5	48	6.9
Triple-Negative	83	9.6	113	16.2

Characteristic	Subcohort (N=859) 15% random sample		Breast Cancer Deaths (N=697) 100% sample	
	N	(%)	N	(%)
**Treatment (yes)[γ]**				
Chemotherapy	381	44.4	469	67.3
Radiation	560	65.2	400	57.4
Endocrine therapy	586	68.2	418	60.0

Abbreviations: ER, estrogen receptor; HER2, human epidermal growth factor receptor 2; PR, progesterone receptor; Pre/Peri, premenopausal/perimenopausal

* Number of Imputed values: subcohort: 32 (3.7%); deaths: 32 (4.6%)

[†] Number of Imputed values: subcohort: 80 (9.3%); deaths: 62 (8.9%)

[‡] Number of Imputed values: subcohort: 63 (7.3%); deaths: 74 (10.6%)

[δ] Number of Imputed values: subcohort: 157 (18.3%); deaths: 155 (22.2%)

[γ] Non-exclusive categories

Supplementary Table 2. Body mass index (BMI) and breast cancer-specific mortality according to breast cancer subtype and menopausal status.

BMI	Cohort	Deaths	HR (95% CI) †	HR (95% CI) ‡
**Premenopausal:**				
Luminal A subtype				
Normal weight	65 (48.2)	38 (38.4)	1.0	1.0
Overweight	43 (31.9)	25 (25.3)	1.2 (0.6-2.4)	0.9 (0.4-2.3)
Obese	27 (20.0)	36 (36.4)	3.2 (1.6-6.2)	2.1 (0.8-5.1)
Luminal B subtype				
Normal weight	16 (55.2)	20 (46.5)	1.0	1.0
Overweight	6 (20.7)	7 (16.3)	2.4(0.4-14.9)	2.6 (0.2-33.1)
Obese	7 (24.1)	16 (37.2)	3.3 (0.8-12.9)	1.5 (0.3-7.5)
Her-2 overexpressing subtype				
Normal weight	6 (66.7)	10 (47.6)	--	--
Overweight	2 (22.2)	5 (23.8)	--	--
Obese	1 (11.1)	6 (28.6)	--	--
Triple Negative subtype				
Normal weight	8 (32.0)	16 (36.4)	1.0	--
Overweight	9 (36.0)	18 (40.9)	3.3 (0.4-26.6)	--
Obese	8 (32.0)	10 (22.7)	1.2 (0.2-7.1)	--
**Postmenopausal:**				
Luminal A subtype				
Normal weight	221 (43.9)	128 (41.4)	1.0	1.0
Overweight	160 (31.8)	77 (24.9)	0.8 (0.6-1.2)	0.6 (0.4-1.0)
Obese	123 (24.4)	104 (33.7)	1.4 (1.0-2.1)	1.2 (0.7-1.9)
Luminal B subtype				
Normal weight	28 (35.9)	24 (28.2)	1.0	1.0
Overweight	26 (33.3)	27 (31.8)	1.0 (0.3-3.0)	1.1 (0.2-5.8)
Obese	24 (30.8)	34 (40.0)	1.8 (0.6-5.5)	1.1 (0.1-8.1)
Her-2 overexpressing subtype				
Normal weight	8 (38.1)	15 (55.6)	--	--
Overweight	7 (33.3)	5 (18.5)	--	--
Obese	6 (28.6)	7 (25.9)	--	--
Triple Negative subtype				
Normal weight	25 (43.1)	28 (40.6)	1.0	1.0
Overweight	20 (34.5)	21 (30.4)	0.9 (0.3-2.5)	0.9 (0.3-3.2)
Obese	13 (22.4)	20 (29.0)	1.3 (0.4-3.7)	1.1 (0.3-3.6)

Abbreviations: CI, confidence interval; HER2, human epidermal growth factor receptor 2; HR, hazard ratio; --, not calculated due to small cell sizes

* Normal weight = 18.5 – 24.9 kg/m^2; overweight = 25.0 – 29.9 kg/m^2; obese = ≥30 kg/m^2

† HR1 - Adjusted for age (10 year age groups) and Hispanic ethnicity.

‡ HR2 – Adjusted for age (10 year age groups), Hispanic ethnicity, stage at diagnosis (I, II, III/ IV) and tumor grade (1, 2, 3/4)

Mediation proportions not calculated due to small cell sizes/unstable estimates.
